# Trehalose metabolism genes of *Aphelenchoides besseyi* (Nematoda: Aphelenchoididae) in hypertonic osmotic pressure survival

**DOI:** 10.1242/bio.023267

**Published:** 2017-04-10

**Authors:** Qiaoli Chen, Danlei Li, Feng Wang, Ruizhi Zhang, Yaming Ling

**Affiliations:** College of Forestry, Northeast Forestry University, Harbin, Heilongjiang, China

**Keywords:** *Aphelenchoides besseyi*, Osmobiosis, Trehalose, Trehalose-6-phosphate synthase, Trehalase

## Abstract

Some organisms can survive extreme desiccation caused by hypertonic osmotic pressure by entering a state of suspended animation known as osmobiosis. The free-living mycophagous nematode *Aphelenchoides besseyi* can be induced to enter osmobiosis by soaking in osmolytes. It is assumed that sugars (in particular trehalose) are instrumental for survival under environmental stress. In *A. besseyi*, two putative trehalose-6-phosphate synthase genes (TPS) encoding enzymes catalyzing trehalose synthesis, and a putative trehalase gene (TRE) encoding enzymes that catalyze hydrolysis of trehalose were identified and then characterized based on their transcriptome. RT-qPCR analyses showed that each of these genes is expressed as mRNA when *A. besseyi* is entering in, during and recovering from osmobiosis, but only for certain periods. The changes of TRE activity were consistent with the transcript level changes of the TRE gene, and the trehalose level declined at certain periods when the nematodes were in, as well as recovering from, osmobiosis; this suggested that the hydrolysis of threhalose is essential. The feeding method of RNA interference (RNAi) was used to temporarily knock down the expression of each of the TPS and TRE genes. No obviously different phenotype was observed from any of the genes silenced individually or simultaneously, but the survival under hypertonic osmotic pressure reduced significantly and the recovery was delayed. These results indicated that trehalose metabolism genes should play a role in osmobiosis regulation and function within a restricted time frame.

## Abstract (precis)

**Summary:** Trehalose metabolism genes should play a role in osmobiosis regulation and also function within a restricted time frame. Silence of any of these genes will cut down the nematode survival under hypertonic osmotic condition.

## INTRODUCTION

Nematodes can turn into a dormant state to survive gradual loss of water (Perry, 1999) when the metabolism is reduced to a level that cannot be detected (Clegg, 2001; Opperman, 2000). If the water loss is caused by evaporative dehydration it is termed ‘anhydrobiosis’ (Crowe et al., 1992), and when the water loss is caused by hypertonic osmotic pressure the nematodes are in ‘osmobiosis’ (Glazer and Salame, 2000; Yan et al., 2011). The rice white tip nematode, *Aphelenchoides besseyi*, is widely distributed throughout almost all rice-growing regions. It causes serious nematode diseases in rice and decreases yield by 10-20% in general, and by over 30% in severe cases (Bridge et al., 1990; Wang et al., 1993). *A. besseyi* also infects other herbaceous crops and numerous ornamental plants (Tsay et al., 1995), which results in large economic losses all over the world (Duncan et al., 2006). *A. besseyi* is able to last for a long time under hypertonic osmotic pressure, which increases the difficulty of preventing and controlling infestations of these nematodes. Thus far, unlike anhydrobiosis, little attention has been devoted to the genetic aspects of osmobiosis. Nevertheless, in a moistened soil environment, *A. besseyi* is constantly exposed to various osmotic conditions and little is known about the ability of *A. besseyi* to withstand osmotic stresses. *A. besseyi* can also be used as a genetic model to unravel the mechanisms of hypertonic osmotic pressure tolerance within metazoans.

Trehalose is considered an important product of metabolic stress, and as an osmoprotectant it is believed to be involved in protecting cell membrane structure and integrity in nematodes (Behm, 1997; Dmitryjuk and Zółtowska, 2004; Erkut et al., 2011; Fairbairn and Passey, 1957; Pellerone et al., 2003; Zoltowska et al., 2002). In early 1975, trehalose was proposed to be important for the desiccation tolerance of nematodes, and this finding was based on the correlation between the amount of trehalose and the ability to survive dehydration (Crowe and Madin, 1975). In most eukaryotes, trehalose-6-phosphate synthase (TPS) (Delorge et al., 2015; Li et al., 2011; Van Houtte et al., 2013) and trehalose-6-phosphate phosphatase (TPP) (Ponnu et al., 2011; Vandesteene et al., 2012; Yadav et al., 2014) are responsible for trehalose synthesis (Pellerone et al., 2003), with trehalase (TRE) catalyzing the hydrolysis of trehalose (Pellerone et al., 2003). By hydrolyzing trehalose, various tissues and organs obtain glucose, which effectively protects somatic cells and allows them to adapt to a stress environment and enhance their resilience (Behm, 1997). Until now, no TPP gene has been identified in nematodes. As TRE has a synergistic effect with hormones and regulates changes in the concentration of sugar (Van Houtte et al., 2013), and is the only hydrolase that specifically hydrolyzes trehalose into glucose, it has been suggested to act as the key enzyme of trehalose metabolism (Dmitryjuk et al., 2006; Pellerone et al., 2003). For these reasons, this study focused on the changes of transcript level of TPS genes and TRE genes as well as TRE activity and trehalose level during osmobiosis.

Anhydrobiosis associated with trehalose and TRE has been studied in a small number of nematode species (Erkut et al., 2011; Goyal et al., 2005). In *Aphelenchus avenae*, anhydrobiosis induction is at least partly caused by the upregulation of trehalose synthase genes (Goyal et al., 2005) and a high concentration of trehalose, equivalent to 10-15% of its dry weight, has been reported during anhydrobiosis (Madin and Crowe, 1975). In *Caenorhabditis elegans*, anhydrobiosis is correlated with a several-fold increase in the amount of trehalose (Erkut et al., 2011). Therefore, TRE genes are supposed to repress whereas TPS genes upregulate by desiccation stress, resulting in the accumulation of trehalose. However, in some desiccation-tolerant invertebrates, such as bdelloid rotifers (Lapinski and Tunnacliffe, 2003; Tunnacliffe et al., 2005) and some tardigrades (Hengherr et al., 2008), disaccharide accumulation is not detectable, but it has also been shown that TRE genes were highly expressed compared to TPS genes in bdelloid rotifer *Adineta vaga* (Hespeels et al., 2015), explaining why trehalose had not been detected in previous studies of bdelloids during desiccation. Hence, it is still necessary to determine whether trehalose and TRE perform protection mechanisms in the process of dehydration.

This study presents our investigations into the osmobiosis of *A. besseyi* and our identification and characterization of TPS and TRE genes in *A. besseyi*. We used RNA interference (RNAi) to silence the expression of these genes in order to simplify the investigation and test the suitability of the corresponding enzymes as new drug targets. We also detailed the TRE activity and trehalose level that are associated with dehydration and rehydration to embody the function of these genes.

## RESULTS

### cDNA cloning and homology analysis

Two putative TPS genes and one TRE gene were identified. *Ab-tps1* contains a coding sequence of 3753 bp, with a 119 bp 5′ untranslated region (UTR) and a 380 bp 3′ UTR featuring a poly A (polyadenylic acid) tail. *Ab-tps2* contains a coding sequence of 1524 bp, with a 33 bp 5′ UTR and a 322 bp 3′ UTR featuring a poly A tail. *Ab-tre* contains a coding sequence of 1791 bp, with a 90 bp 5′ UTR and a 518 bp 3′ UTR featuring a poly A tail.

*Ab-tps1* has considerable similarity to the *Aphelenchoides fragariae* TPS genes (NCBI Accession: JN881460.1, E value=0.0; JN881461.1, E value=0.0; JN881462.1, E value=0.0). *Ab-tps2* has considerable similarity to the *Toxocara canis* TPS gene (NCBI Accession: KHN76157.1, E value=0.0). *Ab-tre* has considerable similarity to the *C. elegans* TRE gene (NCBI Accession: NP_501058.2, E value=0.0) and the *Loa loa* TRE gene (NCBI Accession: EJD73651.1, E value=0.0). The alignment between Ab-tps1 (Fig. S1A), Ab-tps2 (Fig. S2A) and Ab-tre (Fig. S3A) and its closest homologs indicated that the architectures of these proteins sequence were highly conserved.

The maximum likelihood (ML) trees indicated that Ab-tps1 presented a closer orthologous relationship with plant parasitic nematodes *A. fragariae* and *Aphelenchus avenae* (Fig. S1B), which indicated that the evolution of this protein was consistent with species evolution. No orthologs of Ab-tps2 were acquired from plant parasitic nematodes, therefore Ab-tps2 separated into one branch (Fig. S2B). All of the nematode TREs present one-to-one orthologous relationships with *C. elegans*-tre-2 but not with the paralogs *C. elegans*-tre-1, *C. elegans*-tre-3, *C. elegans*-tre-4, and *C. elegans*-tre-5 (Fig. S3B). These patterns indicate that the duplications that generated these TREs occurred before the divergence of *C. elegans* and *A.* *besseyi.*

### Determination of the dehydration and rehydration time

A 90% MgSO_4_·7H_2_O solution is an effective osmolyte that does not damage *A. besseyi* in a certain period of time (Glazer and Salame, 2000). The time needed for *A. besseyi* soaked in a 90% MgSO_4_·7H_2_O solution to reach complete dehydration (shrunken and stationary) and enter osmobiosis was 300 min at 25°C. The bivariate correlation analysis between the dehydration time and the length of dehydrated *A. besseyi* showed a significant correlation (*r*=−0.963), which indicated that there was a significant correlation between the length of *A. besseyi* and the dehydration time.

The time for *A. besseyi* to become motile after being rehydrated was 35 min after being dehydrated by a 90% MgSO_4_·7H_2_O solution for 320 min at 25°C. The bivariate correlation analysis between the rehydration time and the length of *A. besseyi* rehydrated after a 90% MgSO_4_·7H_2_O solution dehydration showed a correlation (*r*=0.888) which indicated that there was a correlation between the length of *A. besseyi* and the rehydration time.

**Fig. 1.**
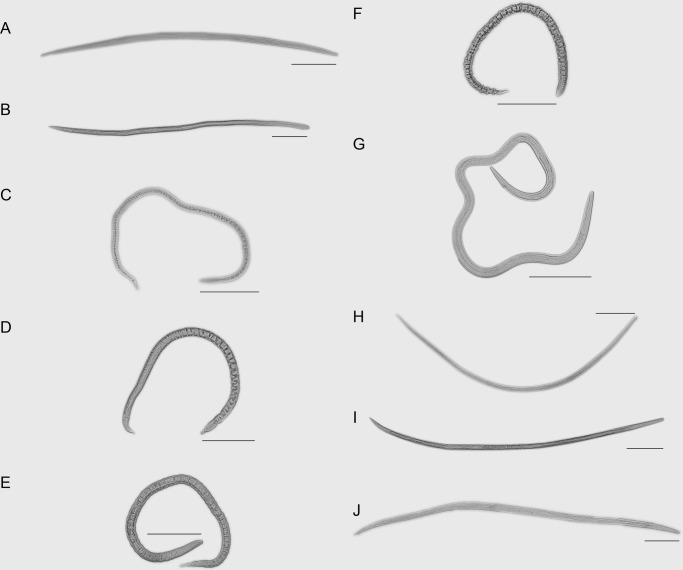
**Stages of dehydration and rehydration.** (A) Stage A, active. (B) Stage B, stiff. (C) Stage C, writhing. (D) Stage D, inching. (E) Stage E, stationary. (F) Stage F, osmobiosis. (G) Stage G, stretching. (H) Stage H, motile. (I) Stage I, twisting. (J) Stage J, revitalized. Scale bars: 100 µm, *n*=20.

Depending on the vitality and altering morphology, the dehydration state as *A. besseyi* entered osmobiosis was divided into 10 time points and the 10 time points were merged into five stages (Table 1, Fig. 1A-E; Fig. S4). In stage A, *A. besseyi* shrinks but has no obvious change in vitality. In stage B, *A. besseyi* shrinks even more and moves stiffly. In stage C, *A. besseyi* continues to shrink and only moves slightly. In stage D, obvious shrinkage does not occur, and *A. besseyi* is nearly stationary. In stage E, *A. besseyi* is stationary and enters osmobiosis.

**Table 1.**
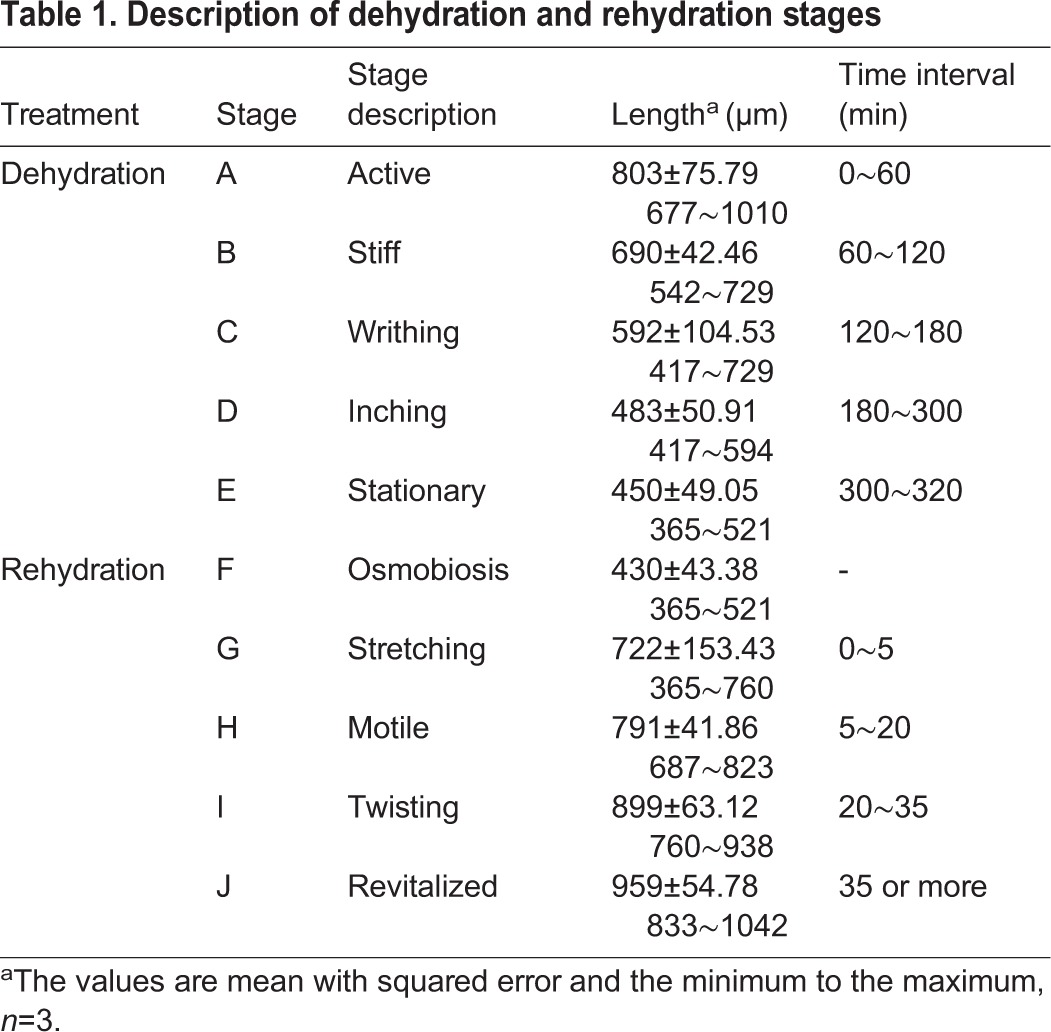
**Description of dehydration and rehydration stages**

The rehydration state as *A. besseyi* recovered from osmobiosis was also divided into five time points and each of it referred to the end of a stage (Table 1, Fig. 1F-J). In stage F, *A. besseyi* is in the osmobiosis state. In stage G, *A. besseyi* has begun stretching but is not motile. In stage H, *A. besseyi* continues stretching and several nematodes begin to twist. In stage I, *A. besseyi* continues stretching and a large number of motile nematodes appear. In stage J, most of the nematodes have stretched to a normal length and regain activity. The Student's *t*-test results indicated that the length of the *A. besseyi* differed significantly from different stages (Table S1).

### Analysis of transcript abundance

**Fig. 2.**
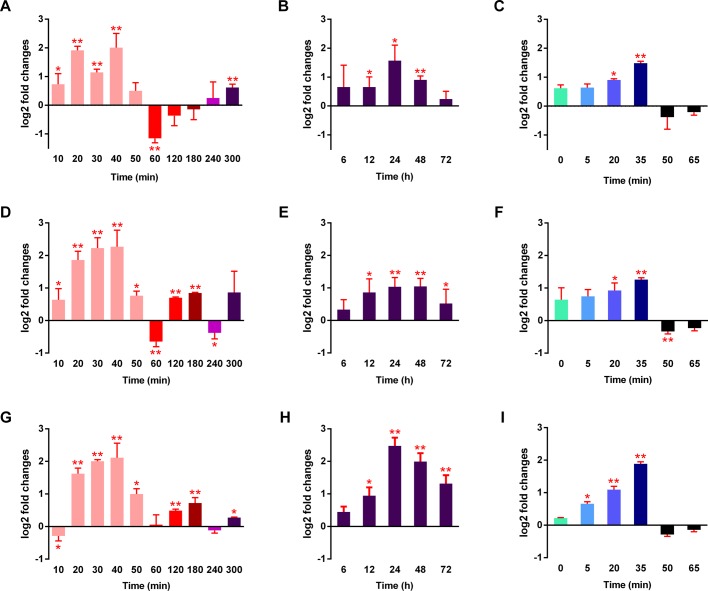
**Analysis of *Ab-tps1*, *Ab-tps2* and *Ab-tre* transcript abundance in *A. besseyi*.** (A) *Ab-tps1* transcript level of *A. besseyi* dehydrated by a 90% MgSO_4_·7H_2_O solution at 10 time points. (B) *Ab-tps1* transcript level of *A. besseyi* under osmobiosis at five time points. (C) *Ab-tps1* transcript level of *A. besseyi* rehydrated in sterile water at six time points after being dehydrated in the 90% MgSO_4_·7H_2_O solution for 320 min. (D) *Ab-tps2* transcript level of *A. besseyi* dehydrated by a 90% MgSO_4_·7H_2_O solution at 10 time points. (E) *Ab-tps2* transcript level of *A. besseyi* under osmobiosis at five time points. (F) *Ab-tps2* transcript level of *A. besseyi* rehydrated in sterile water at six time points after being dehydrated in the 90% MgSO_4_·7H_2_O solution for 320 min. (G) *Ab-tre* transcript level of *A. besseyi* dehydrated by a 90% MgSO_4_·7H_2_O solution at 10 time points. (H) *Ab-tre* transcript level of *A. besseyi* under osmobiosis at five time points. (I) *Ab-tre* transcript level of *A. besseyi* rehydrated in sterile water at six time points after being dehydrated in the 90% MgSO_4_·7H_2_O solution for 320 min. Error bars indicate standard deviation (*P<0.05, **P<0.01, treated nematodes test contrasted nematodes, *n*=3).

After being soaked in a 90% MgSO_4_·7H_2_O solution, the transcripts of *Ab-tps2* and *Ab-tre* increased as the time of dehydration increased from 10 to 40 min (the first half of stage A) and was significantly upregulated in response to dehydration for 20 to 40 min (the middle of stage A), with the highest levels detected at 40 min. Then their transcripts decreased as the time of dehydration increased from 50 to 300 min (the second half of stage A and stages B to the beginning of stage E) and were similar to the control group. During these stages the trend of transcripts for *Ab-tps1* were the same as it for *Ab-tps2* and *Ab-tre* except for 30 min when there was a visible decrease (Fig. 2A,D,G). During the osmobiosis the transcripts of the all three genes gradually increased from 6 h to 24 h and then decreased from 24 h to 72 h (stage E) (Fig. 2B,E,H).

Being rehydrated after dehydration in a 90% MgSO_4_·7H_2_O solution resulted in an increase in the transcript levels of all three genes as the time of rehydration increased from 0 min to 35 min (stages G-I) and appeared to be significantly upregulated in response to a 20 to 35 min rehydration (stage I), with the highest levels detected at 35 min (the end of stage I). The transcripts of these three genes decreased as the time of rehydration increased from 35 min to 50 min (stage J) (Fig. 2C,F,I).

### Changes in trehalase activity and trehalose level during dehydration and rehydration

**Fig. 3.**
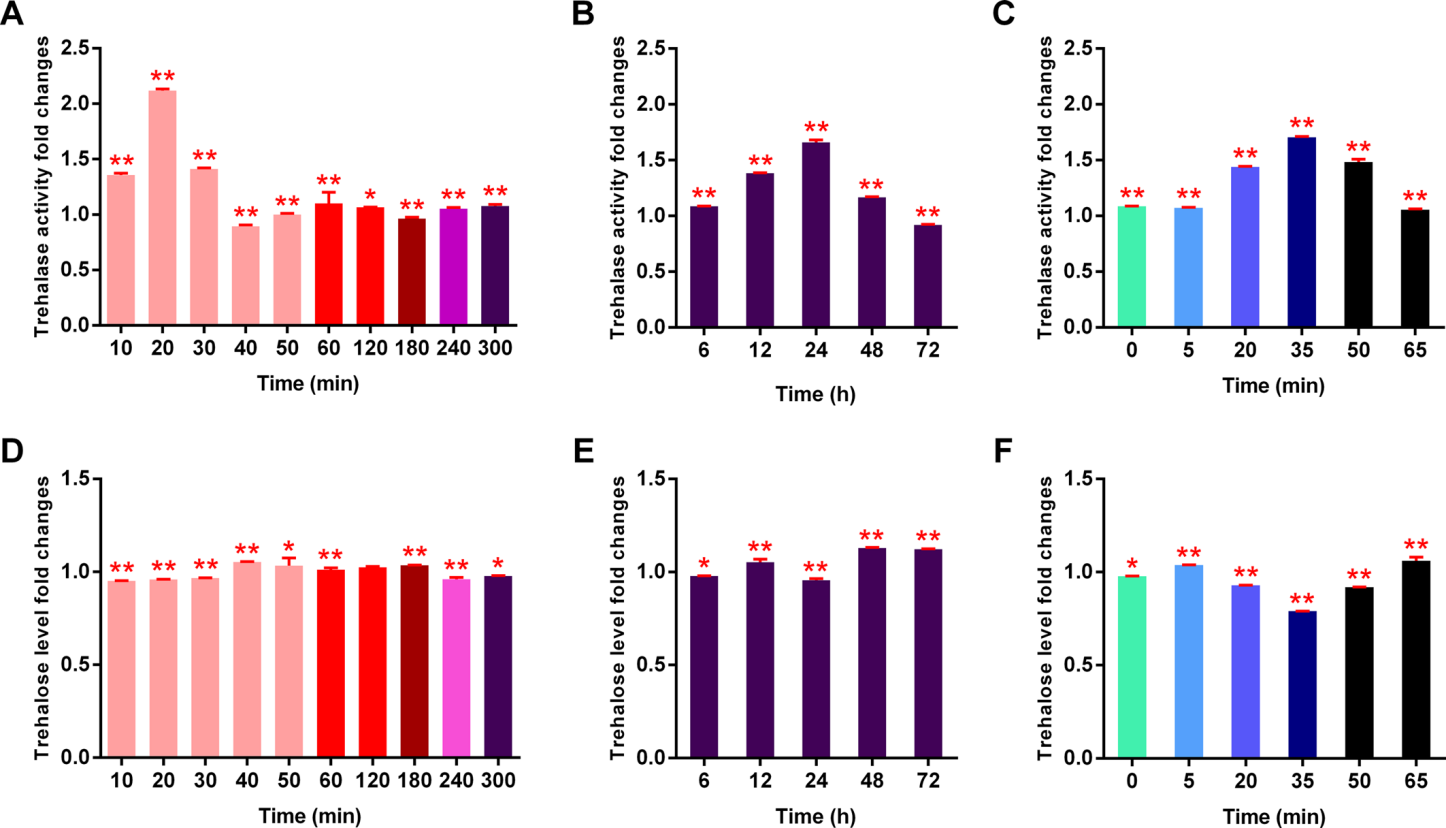
**Analysis of trehalase activity and trehalose level of *A. besseyi*.** (A) trehalase activity of *A. besseyi* dehydrated by a 90% MgSO_4_·7H_2_O solution at 10 time points. (B) Trehalase activity of *A. besseyi* under osmobiosis at five time points. (C) Trehalase activity of *A. besseyi* rehydrated in sterile water at six different time points after being dehydrated in a 90% MgSO_4_·7H_2_O solution for 320 min. (D) Trehalose level of *A. besseyi* dehydrated by a 90% MgSO_4_·7H_2_O solution at 10 time points. (E) Trehalose activity of *A. besseyi* under osmobiosis at five time points. (F) Trehalose level of *A. besseyi* rehydrated in sterile water at six different time points after being dehydrated in a 90% MgSO_4_·7H_2_O solution for 320 min. Error bars indicate standard deviation of mean (*P<0.05, **P<0.01, treated nematodes test contrasted nematodes, *n*=3).

After soaking in a 90% MgSO_4_·7H_2_O solution, the TRE activity was significant higher than the control group as the time of dehydration increased from 10 to 30 min (the first half of stage A) and the highest level appeared at 20 min. The TRE activity was similar to the control group as the time of dehydration increased from 40 to 300 min (the second half of stage A and stage B-E) (Fig. 3A). During the osmobiosis, the TRE activity increased from 6 h to 24 h and decreased for the later three days (Fig. 3B) when the survival was more than a half. Being rehydrated after dehydration in a 90% MgSO_4_·7H_2_O solution resulted in an increase of TRE activity as the time of rehydration increased from 0 min to 50 min (stages G-I and the beginning of stage J) with the highest level appeared at 35 min (the end of stage I); and the TRE activity decreased as the time of rehydration increased from 50 min to 65 min (stage J) (Fig. 3C).

Compared with the control group, the trehalose level was lower as the time of dehydration increased from 10 to 30 min (the first half of stage A) and a little higher as the time of dehydration increased from 40 to 180 min (the second half of stage A and stage B-C). There were reductions in response to dehydration for 240 min (stage D) and for 300 min (the beginning of stage E) (Fig. 3D). However, all the changes of trehalose level from stage A to the beginning of stage E were not significant. During the osmobiosis, the trehalose level obviously decreased from 12 h to 24 h and increased for the later two days (stage E) (Fig. 3E). Being rehydrated after dehydration in a 90% MgSO_4_·7H_2_O solution resulted in a distinct reduction of trehalose level as the time of rehydration increased from 5 min to 50 min (stages H-I and the beginning of stage J). The trehalose level of the rehydrated group was similar to the control group at stage F, stage G and stage J (Fig. 3F).

### RNAi interference

**Fig. 4.**
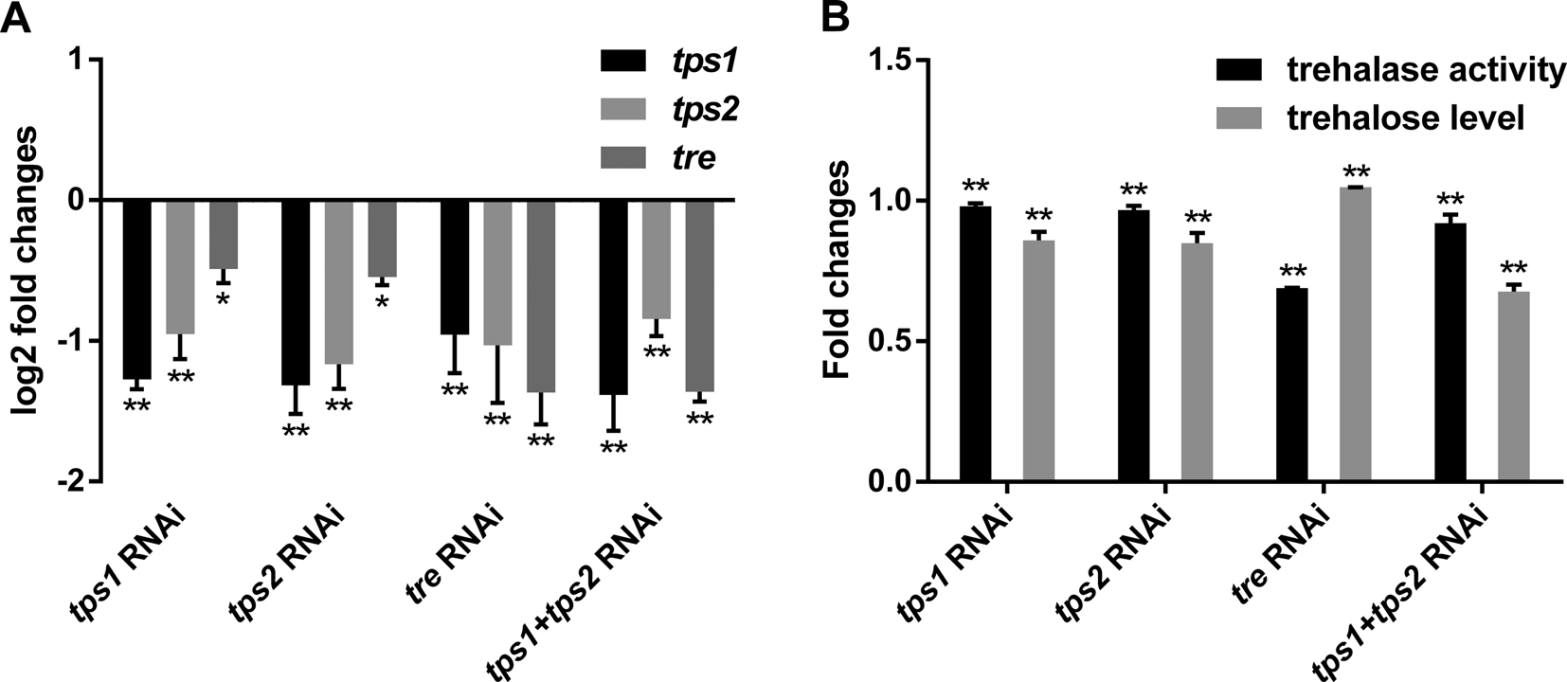
**Transcript abundance, trehalase activity and trehalose level analysis for RNAi-treated *A. besseyi*.** (A) *Ab-tps1*, *Ab-tps2* and *Ab-tre* transcript level log2_(RNAi-treated/RNAi-free)_ fold of *A. besseyi*. (B) Trehalase activity fold of *A. besseyi* (RNAi-treated/RNAi-free) and Trehalose level fold of *A. besseyi* (RNAi-treated/RNAi-free). Error bars indicate standard deviation of mean (*P<0.05, **P<0.01, treated nematodes test contrasted nematodes, *n*=3).

Significant silencing was found in *A. besseyi*. The *Ab-tps1*, *Ab-tps2* and *Ab-tre* dsRNA had no obvious effect on the transcript level of *Ab*-*Actin* (*Ab-28sRNA* as an internal control, using primers Ab-28sRNA-F and Ab-28sRNA-R) (Table S2, Fig. S5); however, whichever TPS gene was silenced would make the transcript of the other TPS gene reduce and the silencing of *Ab-tre* would make the transcripts of both TPS genes reduce, but the transcript level of *Ab-tre* would also be reduced by *Ab-tps1*+*Ab-tps2* silencing (Fig. 4A). Reductions of trehalase activity and trehalose level were found in *Ab-tps1*, *Ab-tps2* and *Ab-tps1*+*Ab-tps2* RNAi-treated *A. besseyi* and a significant reduction of trehalase activity and an increase of trehalose level were found in *Ab-tre* RNAi-treated *A. besseyi* (Fig. 4B), which indicated that RNAi was potent and specific for *A. besseyi* by soaking.

**Fig. 5.**
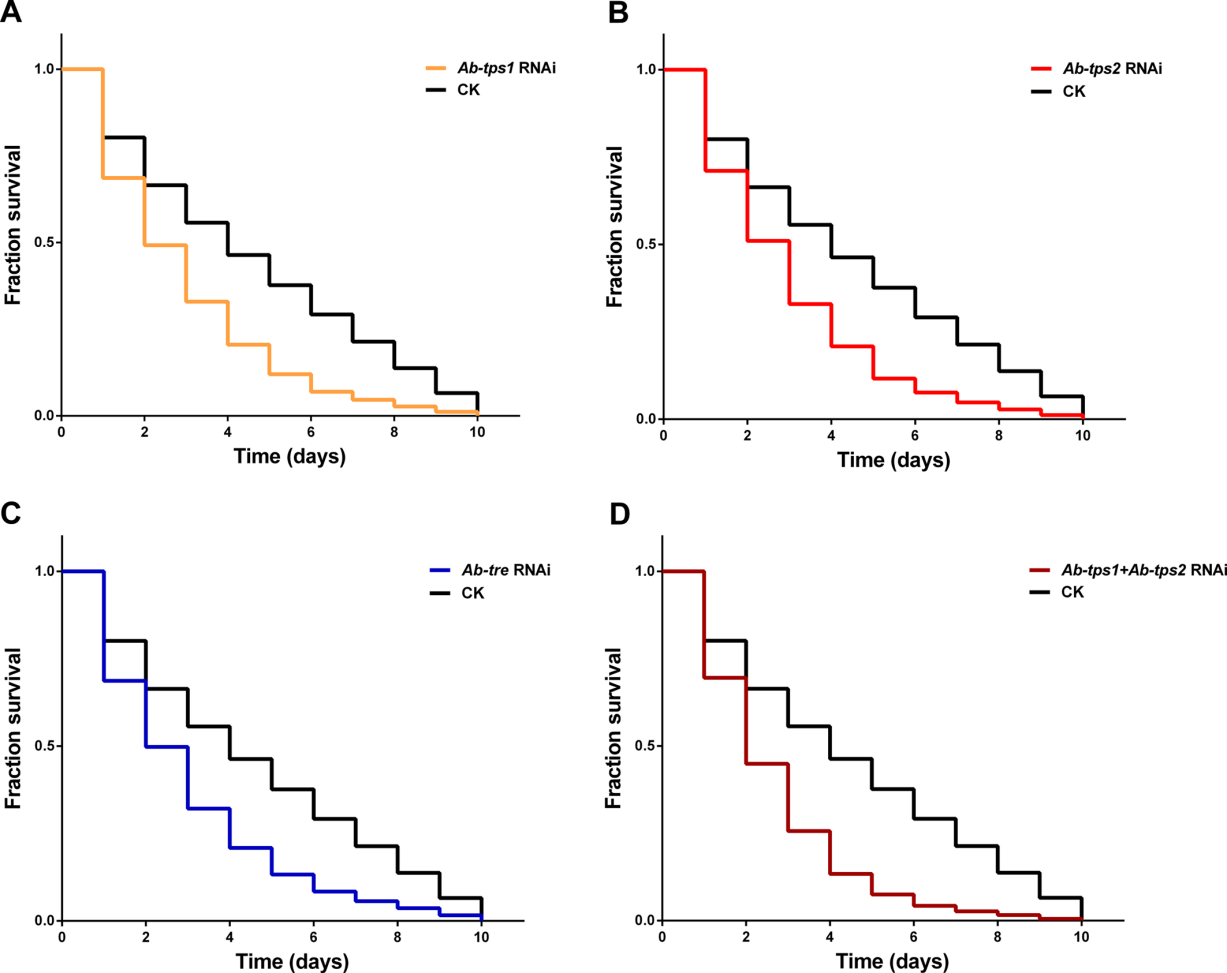
**Survival of *A. besseyi* in sterile water after dehydration in a 90% MgSO_4_·7H_2_O solution for 1 day to 10 days.** (A) *Ab-tps1* RNAi *A. besseyi* and RNAi-free *A. besseyi*. (B) *Ab-tps2* RNAi *A. besseyi* and RNAi-free *A. besseyi*. (C) *Ab-tre* RNAi *A. besseyi* and RNAi-free *A. besseyi*. (D) *Ab-tps1+Ab-tps2* RNAi *A. besseyi* and RNAi-free *A. besseyi* (*n*=3).

**Fig. 6.**
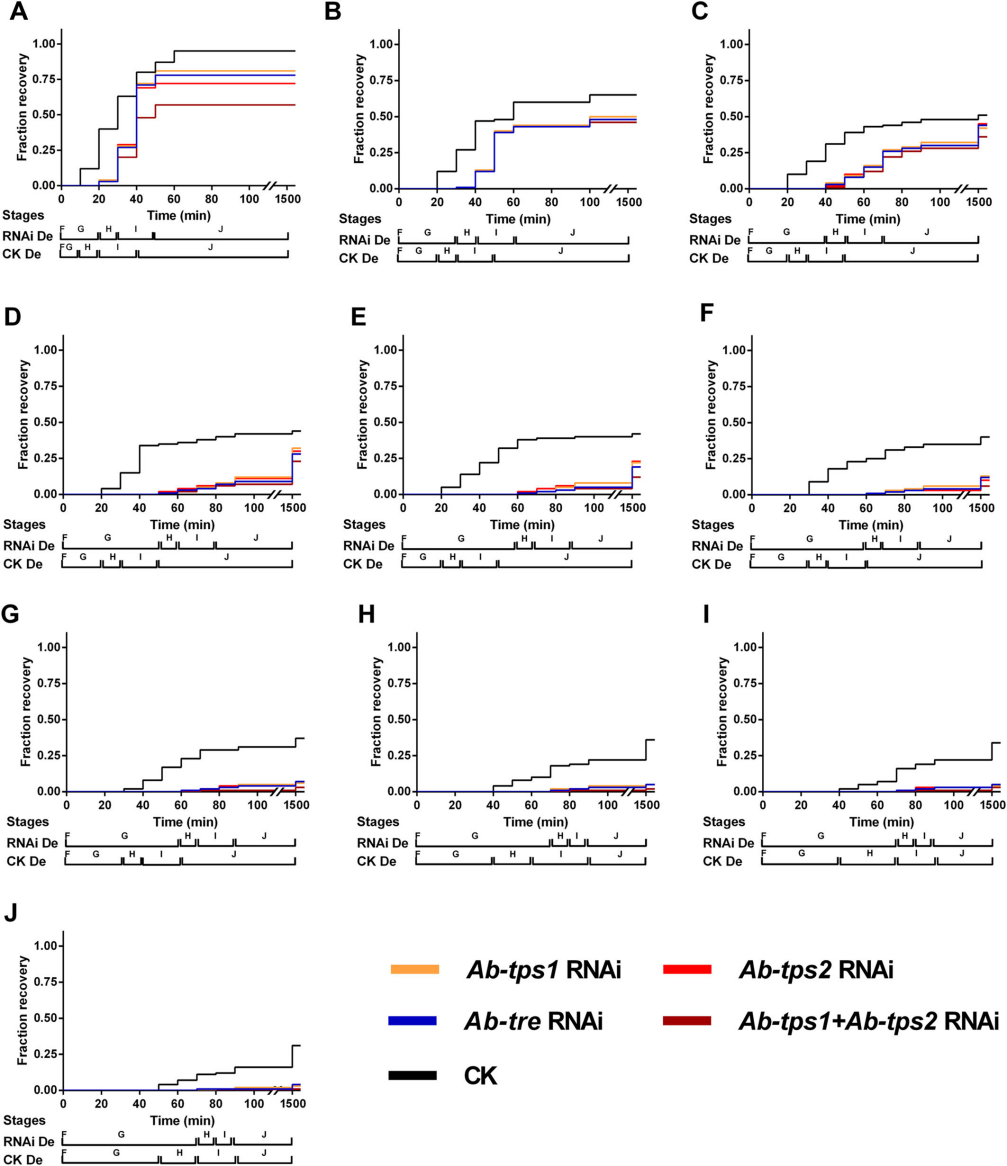
**Recovery of *A. besseyi* in sterile water after dehydration in a 90% MgSO_4_·7H_2_O solution for 1 day to 10 days.** (A) 1 day; (B) 2 days; (C) 3 days; (D) 4 days; (E) 5 days; (F) 6 days; (G) 7 days; (H) 8 days; (I) 9 days; (J) 10 days. F, Stage F; G, Stage G; H, Stage H; I, Stage I, J, Stage J (*n*=3).

All the RNAi groups (RNAi-treated nematodes) presented a survival rate of more than 99% for the whole 10-day study, which is not significantly different to that of the CK group (RNAi-free nematodes). There was no significant difference over survival rate, morphology and vitality between all the RNAi-De groups (dehydrated RNAi-treated nematodes) except for the *Ab-tps1+Ab-tps2* RNAi-treated group, which showed a slightly bigger reduction of survival (Fig. S6). However, compared to the RNAi-De groups, the survival rate of the CK-De group (dehydrated RNAi-free nematodes) reduced significantly more slowly from 1 day to 10 days (Fig. 5), and, during rehydration, stages H and I of the RNAi-De groups were delayed compared with that of the CK-De group (Fig. 6).

The Student's *t*-test results indicated that the survival rate of the RNAi-De groups differed from that of the CK-De group (Table S3). The bivariate correlation analysis between the dehydration time and the survival rate of the RNAi-De groups showed correlations (*r_tps1_*=−0.925, *r_tps2_*=−0.939, *r_tre_*=−0.914 and *r_tps1_*_+*tps2*_=−0.927), as well as the CK-De group (*r*=−0.846).

## DISCUSSION

In this study, we examined the transcript level of *Ab-tps1*, *Ab-tps2* and *Ab-tre* mRNA. Our study showed that the function of these three genes was related to osmobiosis as they influenced the resumption of vitality and the survival rate of the nematodes, and responded positively to hypertonic osmotic pressure within a restricted time frame.

Before nematodes enter osmobiosis, they experience a dehydration process causing gradual changes in vitality and morphology of the nematodes (Glazer and Salame, 2000) and these changes can be divided into five stages. When dehydrated nematodes are immersed in water they undergo a lag period related to the severity of desiccation (Wharton and Aalders, 1999) and present considerable morphological changes (Opperman, 2000; Wharton et al., 1985). The presence of a lag period suggests that certain repair or restoration processes must be undertaken before normal nematode activity can resume (Wharton and Aalders, 1999). Increased exposure to desiccation increases the length of the lag phase or may cause death of nematodes. Thus, an accumulation of damages may occur over time when osmobiosis nematodes remain in a dehydrated state, and this accumulation may also influence gene transcription mechanisms. Hence, we focused on researching the recovery of the nematode from the early osmobiosis stage to eliminate the accumulation of damages.

Trehalose has been suggested to play a role in protecting membranes and proteins by replacing structural water or by forming a stabilizing intracellular glass (Crowe et al., 1987) as well as maintaining membrane fluidity (Crowe et al., 1984). Therefore, TRE genes are suggested to be repressed, whereas TPS genes can be upregulated by hypertonic osmotic pressure to result in the accumulation of trehalose when nematodes are under hostile environments. We examined the transcription mechanisms of every specific phase and found out that *Ab-tps1*, *Ab-tps2* and *Ab-tre* presented a dehydration or rehydration response function. Nonetheless, differences occurred in the transcript levels of these three genes at different dehydration stages with the highest expression appearing in the middle of stage A, whereas significant transcript level changes did not occur in other stages. The upregulations of transcripts occurred from 20 to 40 min when there was no obvious change in vitality, although the nematodes had begun to shrink (Fig. S4). During the period of rehydration from osmobiosis, the upregulations of transcription occurred only at stage I, in which a large number of motile nematodes began twisting and stretched nearly to their normal length. However, the largest contraction of nematodes during dehydration occurred at the beginning of stages A and C and the nematodes stretched the most at stage G when the transcript levels were almost unchanged (Fig. S4, Table S1). Thus, these three genes may not function in the morphological changes of the nematodes. Interestingly, these observations also indicated that the upregulations of the transcript of all these three genes only appeared in the middle of stage A, a certain period of stage E and at the end of stage I, suggesting that they may only function at the entrance to a certain period during osmobiosis and the recovery from osmobiosis. Our results also indicate that the severity of osmotic pressure may influence the time required to induce osmobiosis or resume active metabolism.

In our study, during the entrance of osmobiosis, the TRE activity increased significantly in the first part of stage A and kept at an almost stable state for the later stages (the second half of stage A to the beginning of stage E) for a while. Then during the osmobiosis (stage E) the TRE activity increased again and later decreased (Fig. 3B). During all these periods the trehalose level declined in accompaniment with, but not as significantly as, the increase of TRE activity suggesting that there may have been a process of constant accumulation as well as the consumption of trehalose during the adaption of osmobiosis. In spite of the unobvious declines of trehalose level appearing at those stages, there was a significantly low level shown at the end of stage I which suggested that by being hydrolyzed by TRE, trehalose of *A. besseyi* mostly functions at the beginning of the encounter of hypertonic osmotic pressure, and when the nematode is under osmobiosis for a certain time, as well as when the nematode is recovering from osmobiosis, but all under specific periods.

The changes of TRE activity were not only consistent with the changes of the transcripts of *Ab-tre*, but also *Ab-tps1* and *Ab-tps2* which might account for the indistinctive changes of trehalose; however the level of trehalose still declined as long as the transcript of *Ab-tre* and TRE activity increased which indicated that trehalose might be more likely to be effective by being hydrolyzed, and this also testified that TRE and trehalose should play a role in coping with hypertonic dehydration. As the changes of trehalose level may be impacted by TRE as well as TPS, the ability of trehalose in response to environmental stress should be further validated at different time points. This may explain why trehalose had not been detected in previous studies of bdelloids (Lapinski and Tunnacliffe, 2003; Tunnacliffe et al., 2005) and some tardigrades (Hengherr et al., 2008) suffering anhydrobiosis.

We dehydrated and maintained the nematodes in osmobiosis for 0 min to 10 days (Reardon et al., 2010; Wharton and Aalders, 1999) to examine the function of *Ab-tps1*, *Ab-tps2* and *Ab-tre* using the RNAi technique. In *C. elegans*, if any of the TRE and TPS genes were silenced individually or in groups, obvious phenotypes were not observed and the recovery from environmental stress was not affected (Pellerone et al., 2003). Our results also showed that obvious phenotype changes did not occur between the RNAi-treated nematodes and the control nematodes. Nevertheless, there is a feedback mechanism between these three genes. The silence of each TPS gene impacted more on the other TPS gene than the *Ab-tre*, but silencing both TPS genes would cut down the transcript level of *Ab-tre*, although a silence of *Ab-tre* caused the transcript of both TPS genes to decline. This was consistent with the former result that the hydrolysis of trehalose may be more essential and also indicated that *Ab-tre* may play a leading role in trehalose metabolism.

Although *Ab-tps1*, *Ab-tps2*, *Ab-tre* and *Ab-tps1+Ab-tps2* silencing may not have an effect on the lifespan of normal nematodes, being compared with the control nematodes, the survival of the osmobiosis nematodes subjected to RNAi treatment decreased significantly (Fig. S6, Table S3). The gap between the RNAi-De groups and the CK-De group became bigger over the first 5 days and trailed off a little over the next 5 days, indicating that after a certain duration of exposure to hypertonic osmotic pressure, the reaction of *Ab-tps1*, *Ab-tps2* and *Ab-tre* significantly affected the survival rate of the nematodes. During rehydration, the time for stage H and I to appear were delayed for the RNAi-treated nematodes. Stage H is the stage at which the nematodes begin to recover, and a higher number of recovered nematodes appeared in stage I, which could represent the ability of the nematodes to recover. Thus even though *Ab-tps1*, *Ab-tps2* and *Ab-tre* may not function in the morphological changes to the nematode, they may function in osmobiosis regulation and the onset of stages H and I during rehydration, and also influence the survival rate of the nematodes. These results once again attested that *Ab-tps1*, *Ab-tps2* and *Ab-tre* are associated with osmobiosis.

To conclude, this study shows that *Ab-tps1*, *Ab-tps2* and *Ab-tre* are involved in osmobiosis. They help nematodes to adapt to hypertonic osmotic pressure and are positive effectors of osmobiosis. Our results highlight the importance of trehalose metabolism genes in the hypertonic osmotic pressure tolerance and reveal the potential for using gene silencing technology to control rice white tip nematodes. Further investigations of genes involved in osmobiosis will shed light on the molecular principles underlying osmobiosis regulation and provide useful methods for controlling *A. besseyi*.

## MATERIALS AND METHODS

### Nematodes and DNA sequencing

The nematode *A. besseyi* (NCBI BioSample accession No. SAMN02420038) was cultured on *Botrytis cinerea* at 25°C in the dark. A Baermann funnel was used to extract the nematodes (male, female, and juvenile mixed together at the ratio of 1:2:1) which were then frozen in a mortar with liquid nitrogen and powdered using a pestle. Total RNA was extracted from the powder using TRIzol (Invitrogen, USA, cat. no. 15596-026) (Wang et al., 2016, 2012). Then the Promega AMV reverse transcription system was used (Promega, USA, cat. no. A3500) according to the manufacturer's instructions and Oligo (dT)_18_ was used as a primer for the first chain of cDNA.

Based on the transcriptome of *A. besseyi* (Wang et al., 2014), homologs of TPS genes and TRE genes homologs in *A. besseyi* were searched using a numerical algorithm in HMMER (v3.1b2) (Mistry et al., 2013) according to the threshold E-value<10^−10^. The primer pairs of tre-ORF (Ab-tre-ORF-F and Ab-tre-ORF-R), tps1-ORF (Ab-tps1-ORF-F and Ab-tps1-ORF-R) and tps2-ORF (Ab-tps2-ORF-F and Ab-tps2-ORF-R) (Table S2) were designed to amplify the cDNA fragments that cover the open reading frame (ORF) of these genes. Positive clones were sent to Sangon Biotech (Shanghai, China) for sequencing.

### Sequence analysis, alignment and phylogenetic studies

Sequence homology comparisons were conducted using BLASTX and BLASTN searches (http://blast.ncbi.nlm.nih.gov/Blast.cgi). The amino acid sequences of TPS1 from *A. besseyi* and six additional organisms (*A. fragariae*, *A. avenaae*, *C. elegans*, *Anisakis simplex*, *Ascaris suum* and *Toxocara canis*), TPS2 from *A. besseyi* and six additional organisms (*C. elegans*, *A. suum*, *A. simplex*, *T. canis*, *Wuchereria bancrofti* and *Brugia malayi*) and TRE from *A. besseyi* and five additional organisms (*Loa loa*, *A. simplex*, *C. elegans*, *Necator americanus* and *C. brenneri*) were aligned. Based on the amino acid sequences of *A. besseyi* and the additional proteins, ML phylogenetic trees were constructed by using the ML method in MEGA 7.0.21 (Molecular Evolutionary Genetics Analysis, USA).

### Determination of the dehydration and rehydration stages

To determine the dehydration/rehydration stages, the nematodes were separately soaked in osmolyte or rehydrated in water at 25°C. For the dehydration treatment, the nematodes were soaked in a 90% MgSO_4_·7H_2_O solution (Glazer and Salame, 2000). For the rehydration treatment, fully dehydrated nematodes (shrunken and stationary) were soaked in sterile water. The dehydration or rehydration stages were determined according to the organism's morphology and vitality. For each time point, the lengths of 20 nematodes were photographed using a microscope Bx51 (Olympus, Japan). Then the lengths of the nematodes in the pictures were measured by flexible ropes and converted to the real lengths. Three biological replicates were performed in these experiments. A paired-sample Student's *t*-test was used to determine the difference between each time point and each stage. A bivariate correlation analysis (SPASS 13.0) was used to determine how the time of dehydration or rehydration influences the length of *A. besseyi.*

### Analysis of transcript abundance

The transcript level of *Ab-tps1*, *Ab-tps2* and *Ab-tre* under dehydration/rehydration conditions was measured by RT-qPCR using a GoTaq 2-Step RT-qPCR System Kit (Promega, USA, cat. no. A6010) and Stratagene Mx3000P qPCR system (Agilent, USA). The RT-qPCR results were normalized as log_2_ (dehydration or rehydration gene copy/control gene copy)-fold changes with a constitutively expressed gene, *Ab-Actin* as an internal control (using primers Ab-Act-Q-F and Ab-Act-Q-R, Table S2) and *Ab-tps1-Q* (using primers Ab-tps1-Q-F and Ab-tps1-Q-R, Table S2), *Ab-tps-Q* (using primers Ab-tps2-Q-F and Ab-tps2-Q-R, Table S2) and *Ab-tre-Q* (using primers Ab-tre-Q-F and Ab-tre-Q-R, Table S2) as the reference gene.

For the dehydration stages, the nematodes were soaked in a 90% MgSO_4_·7H_2_O solution as the test. For the rehydration stage, fully dehydrated nematodes (shrunken and stationary) from the dehydration state were soaked in sterile water. The control groups consisted of nematodes treated with sterile water for the same amount of time. Three biological replicates were performed in these experiments. A paired-sample Student's *t*-test was used to determine the difference between each time point and each stage.

### Trehalase activity and trehalose level determination

Trehalase activity was determined under dehydration/rehydration and *Ab-trehalase* RNAi conditions using the Trehalase Determination Kit (Cominbio, China, cat. no. HTM-2-Y), BCA Method of Protein Content Kit (Cominbio, China, cat. no. BCAP-2-W) and GeneQuant 1300 ultraviolet spectrophotometer (Biochrom Ltd., UK). Trehalose level was measured using the Trehalose Content Kit (Cominbio, China, cat. no. HT-2-Y) and GeneQuant 1300 ultraviolet spectrophotometer at the same time points.

For the dehydration stages, the nematodes were soaked in a 90% MgSO_4_·7H_2_O solution. For the rehydration stage, fully dehydrated nematodes (shrunken and stationary) were soaked in sterile water. The control groups consisted of nematodes treated with sterile water for the same amount of time. Three biological replicates were performed in these experiments. A paired-sample Student's *t*-test was used to determine the difference between each time point and each stage.

### RNA interference

RNA interference (RNAi) was performed using nematodes at mixed developmental stages as outlined by Urwin et al. (Urwin et al., 2002; Wang et al., 2012). Double-stranded RNA (dsRNA) corresponding to *Ab-tps1* (using two primer pairs Ab-T7-tps1-F/Ab-tps1-iR and Ab-tps1-iF/Ab-T7-tps1-R, Table S2), *Ab-tps2* (using two primer pairs Ab-T7-tps2-F/Ab-trps2-iR and Ab-tps2-iF/Ab-T7-tps2-R, Table S2) and *Ab-tre* (using two primer pairs Ab-T7-tre-F/Ab-tre-iR and Ab-tre-iF/Ab-T7-tre-R, Table S2) were prepared using the MAXIscript T7/T3 RNA Synthesis Kit (Ambion, Japan, cat. no. AM1324M). The RNAi nematodes were soaked in M9 buffer with 10 mM octopamine and dsRNA (3 mg/ml). The control nematodes were soaked in M9 buffer with 10 mM octopamine. DsRNA feeding experiments were performed for each gene individually and in combination with the others to determine whether the TPS and the TRE genes have specific unique functions. After being soaked for 12 h with intermittent stirring at 25°C, the nematodes were thoroughly washed with sterile water to remove the external dsRNA. RT-qPCR experiment was performed to provide the evidence of knockdown of gene expression.

The RNAi-treated and the control nematodes were divided into four groups. The first group, named RNAi, was used to assess the survival of non-dehydrated RNAi-treated nematodes. The second group, RNAi-De, was used to assess the recovery of dehydrated RNAi-treated nematodes. The third group, CK (control check), was used to assess the survival of the non-dehydrated RNAi-free nematodes. The fourth group, CK-De, was used to assess the recovery of the dehydrated RNAi-free nematodes. The dehydrated groups were soaked in a 90% MgSO_4_·7H_2_O solution for 0 min to 10 days, and the non-dehydrated groups were soaked in sterile water for the same amount of time. The recovery of the dehydrated nematodes and the survival of non-dehydrated nematodes in sterile water was monitored daily, and each treatment was repeated three times. A paired-sample Student's *t*-test was used to determine the difference between the RNAi-treated nematodes and the CK nematodes and a bivariate correlation analysis were used to determine how *Ab-trehalase* silencing influenced the recovery of nematodes from osmobiosis.
